# Auxin is involved in arbuscular mycorrhizal fungi-promoted tomato growth and *NADP-malic enzymes* expression in continuous cropping substrates

**DOI:** 10.1186/s12870-020-02817-2

**Published:** 2021-01-18

**Authors:** Yu Wang, Wenze Zhang, Weikang Liu, Golam Jalal Ahammed, Wenxu Wen, Shirong Guo, Sheng Shu, Jin Sun

**Affiliations:** 1 College of Horticulture, Nanjing Agricultural University, Nanjing, 210095 China; 2 College of Horticulture and Plant Protection, Henan University of Science and Technology, Luoyang, 471023 China

**Keywords:** Tomato, Arbuscular mycorrhizal fungi, Continuous cropping substrate, IAA, Root activity

## Abstract

**Background:**

Despite significant limitations of growth medium reuse, a large amount of organic substrate is reused in soilless cultivation of horticultural crops in China. Arbuscular mycorrhizal fungi (AMF) can promote nutrient absorption and improve plant tolerance to biotic and abiotic stresses. However, the mechanisms governing the effects of AMF on crop growth in organic continuous cropping substrates have not been elucidated.

**Results:**

In this study, we showed that the inoculation of AMF in continuous cropping substrates promoted growth and root development, and increased the root and NADP-malic enzyme (NADP-ME) activity of tomato seedlings. Root transcriptome analysis demonstrated that the plant hormone signal transduction pathway was highly enriched, and 109 genes that positively correlated with the AMF-inoculated plant phenotype were obtained by gene set enrichment analysis (GSEA), which identified 9 genes related to indole acetic acid (IAA). Importantly, the levels of endogenous IAA in tomato seedlings significantly increased after AMF inoculation. Furthermore, the application of AMF significantly increased the expression levels of *NADP-ME1* and *NADP-ME2*, as well as the activity of NADP-ME, and enhanced the root activity of tomato seedlings in comparison to that observed without inoculation of AMF. However, these effects were blocked in plants treated with 2,3,5-triiodobenzoic acid (TIBA), a polar transport inhibitor of IAA.

**Conclusions:**

These results suggest that IAA mediates the AMF-promoted tomato growth and expression of *NADP-MEs* in continuous cropping substrates. The study provides convincing evidence for the reuse of continuous cropping substrates by adding AMF as an amendment.

**Supplementary Information:**

The online version contains supplementary material available at 10.1186/s12870-020-02817-2.

## Background

Soilless cultivation promotes crop growth and yield by providing the most suitable environment for root growth and development [1]. Due to the numerous advantages of organic substrate cultivation, such as improved crop yield and quality, reduced pesticide, water and fertilizer use, simple operation and management, reduced investment cost for equipment, avoidance of soil continuous cropping obstacles, and suitability for green organic cultivation, this type of soilless cultivation has been widely practiced and currently accounts for over 75% of the total area of soilless cultivation in China [2]. The annual production and sales volume of organic substrates in China is approximately 10 million m^3^. At the same time, the same volume of the substrates used is produced every year. However, the physical and chemical properties of the used substrates deteriorate when reused, resulting in larger bulk density and lower pH, which are not conducive to crop growth and development [3]. Studies have shown that the numbers of beneficial fungi decrease and those of harmful fungi increase in continuous cropping substrates [4–6]. In addition, the effective nutrient content, substrate enzyme activity, and root index in the rhizosphere environment significantly decrease with the increase in continuous cropping years [4, 5]. However, it is imperative to improve and reuse substrates to reduce the labor input and production costs. At present, few methods have been used to improve continuous cropping substrates, such as substrates disinfection and crop rotation [4, 6]. Interestingly, the inoculation with beneficial microorganisms in the continuous cropping soil has been demonstrated to be an effective way to overcome continuous cropping obstacles [7]. However, the role of beneficial microorganisms in continuous cropping substrates is largely unknown.

Arbuscular mycorrhizal fungi (AMF) are ubiquitous soil microorganisms and obligate symbionts that are important components of terrestrial ecosystems and can form a mutually beneficial symbiosis with approximately 80% of vascular plant roots [8]. The glycoproteins produced by AMF hyphae can create a suitable rhizosphere environment and better conditions for the growth of almost all vascular plants [9]. AMF symbiosis not only increases the input of root carbon but also enhances carbon fixation in soil by reducing the decomposition of soil organic matter and the rhizosphere priming effect [10]. AMF endow soil and roots with a direct connection, enhance plant mineral nutrition, water acquisition and photosynthesis, and alleviate the adverse effects of abiotic stresses, such as heavy metals and drought stress [11–13]. Our previous study showed that AMF inoculation on seedling substrates can reduce the harm of saline-alkali land exerted on processing tomato [14]. In addition, the application of AMF can effectively overcome the continuous cropping obstacles in continuous cropping soil, to promote growth and development of many crops, such as pepper [15], soybean [16], and cucumber [17]. However, the associated mechanism of AMF-promoted growth in continuous cropping systems has not been elucidated.

In this study, we investigated the effect and mechanism of AMF on the cultivation of tomato in continuous cropping organic substrates. The results showed that the inoculation of AMF in the continuous cropping substrates promoted the growth and increased the activity of root and NADP-malic enzyme (NADP-ME), and the level of indole acetic acid (IAA) in tomato seedlings. However, these effects were compromised when plants were treated with IAA polar transport inhibitor, indicating that IAA played a critical role in AMF-promoted plant growth. The results provide a theoretical and practical basis for the cultivation of tomato in continuous cropping substrates.

## Results

### Effects of AMF on root morphology, plant growth and net photosynthetic rate in tomato seedlings

**Fig. 1:**
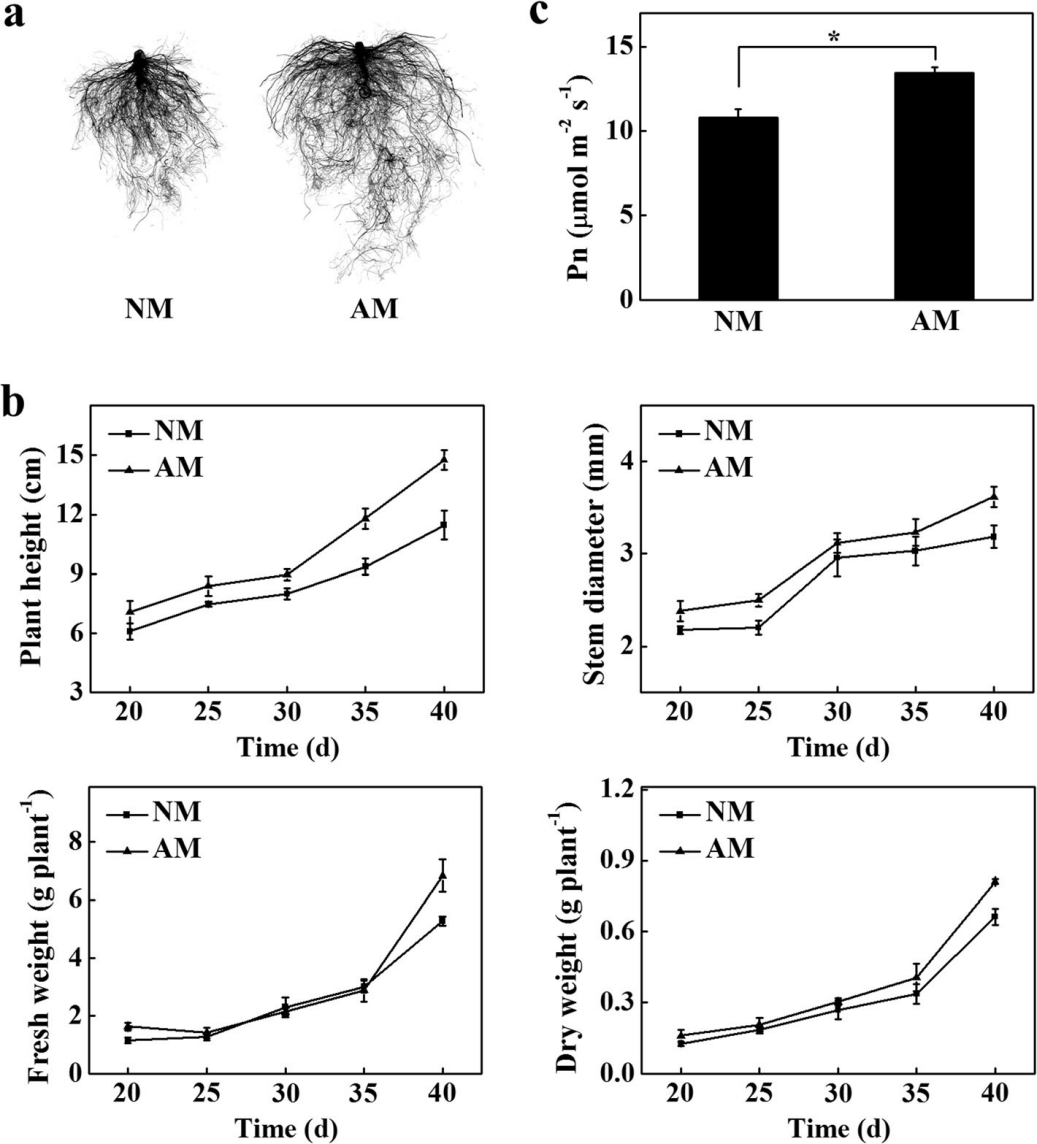
Arbuscular mycorrhizal fungi (AMF) promoted growth of tomato seedlings in the continuous cropping substrates. **a** The root morphology of NM and AM seedlings at 40 d. **b** The plant height, stem diameter, fresh and dry weight of NM and AM seedlings. **c** The net photosynthetic rate (Pn) of NM and AM seedlings at 40 d. The results represent the means ± SE. Three independent experiments were performed, with similar results. * represent significant difference. NM, tomato seedlings cultivated in continuous cropping substrate. AM, tomato seedlings cultivated in continuous cropping substrate inoculation with AMF

To test the role of AMF in continuous cropping substrates, we first compared the growth of tomato in continuous cropping substrates with or without inoculation of AMF. AMF successfully colonized tomato roots, and the colonization rate was approximately 32% in tomato seedlings cultivated in the continuous cropping substrate inoculation with AMF (AM) (Additional file 1: Figure S1). The inoculation of AMF significantly promoted the root growth compared with the tomato seedlings cultivated in the continuous cropping substrate (NM) (Fig. 1a). The root length, total root surface area, total root volume, average root diameter, and the number of root tips of AM seedlings increased by 32, 30, 23, 6, and 27%, respectively, and the fresh weight and dry weight of roots increased by 92 and 54%, respectively, compared with those of the NM seedlings (Additional file 2: Table S1). The plant height, stem diameter, fresh weight and dry weight of AM seedlings increased by 29, 14, 30, and 23%, respectively, at 40 d in comparison to those observed for the NM seedlings (Fig. 1b). Furthermore, the net photosynthetic rate (Pn) and yield of AM seedlings were higher than those observed for the NM seedlings (Fig. 1c; Additional file 3: Figure S2). The results showed that the inoculation of AMF significantly promoted the growth of tomato seedlings in continuous cropping substrates.

### Effects of AMF on root activity and NADP-dehydrogenase activity

**Fig. 2:**
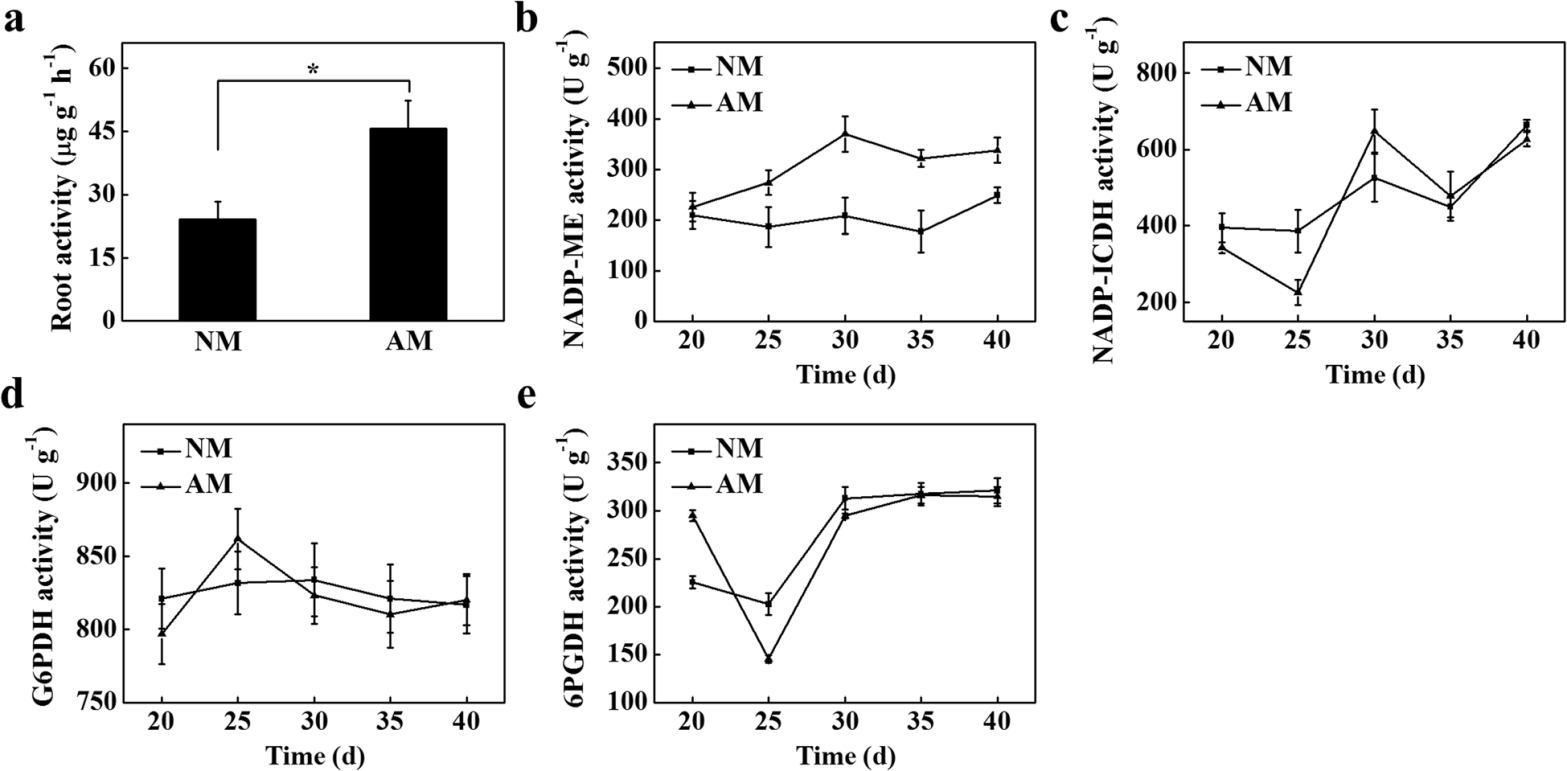
Effects of arbuscular mycorrhizal fungi (AMF) inoculation on the root and NADP-dehydrogenase activity of tomato seedlings. **a** The root activity of NM and AM seedlings at 40 d. **b** The activity of NADP-ME in roots of NM and AM seedlings. **c** The activity of NADP-ICDH in roots of NM and AM seedlings. **d** The activity of G6PDH in roots of NM and AM seedlings. **e** The activity of 6PGDH in roots of NM and AM seedlings. The results represent the means ± SE. Three independent experiments were performed, with similar results. * represent significant difference. NM, tomato seedlings cultivated in continuous cropping substrate. AM, tomato seedlings cultivated in continuous cropping substrate inoculation with AMF

AMF have been shown to improve root development in plants under adverse conditions [18, 19]. Furthermore, the activity of NADP-dehydrogenase is closely related to the root activity of plants [20]. Therefore, we measured the root activity of NM and AM seedlings at 40 d and the NADP-dehydrogenase activity of tomato roots at 20, 25, 30, 35, and 40 d after treatment. The root activity of AM seedlings was 90% higher than that of NM seedlings (Fig. 2a). Furthermore, the NADP-ME activity of AM seedlings was significantly higher than that of NM seedlings (Fig. 2b). However, there was no significant difference in the activity of NADP-isocitrate dehydrogenase (NADP-IDCH), glucose-6-phosphate dehydrogenase (G6PDH), or 6-phosphate gluconate dehydrogenase (6PGDH), between NM and AM seedlings (Fig. 2c-e).

### Transcriptome analysis

To further investigate how AMF increased root activity, we used transcriptome analysis to identify the differentially expressed genes (DEGs) in the roots between NM and AM seedlings. Illumina sequencing was performed on 6 root cDNA libraries (NM and AM treatments, each with 3 biological replicates) after cultivation of tomato seedlings for 30 d. After removing the low-quality reads, we obtained 397,526,276 clean reads (Additional file 4: Table S2). The average values of Q20, Q30, and clean read rate of the three NM libraries were 98, 92, and 92%, and these indexes of AM libraries were 98, 92, and 93%, respectively (Additional file 4: Table S2). Over 92% of the reads matched the tomato genome, and more than 90% of the reads were uniquely mapped (Additional file 5: Table S3). The clustering heat map intuitively showed the gene expression of NM and AM with relatively consistent and good repeatability (Additional file 6: Figure S3). Moreover, there were 4522 genes with significant differences between NM and AM, including 2566 downregulated genes and 1956 upregulated genes (Additional file 7: Table S4).

### Gene ontology (GO) enrichment analysis

**Fig. 3:**
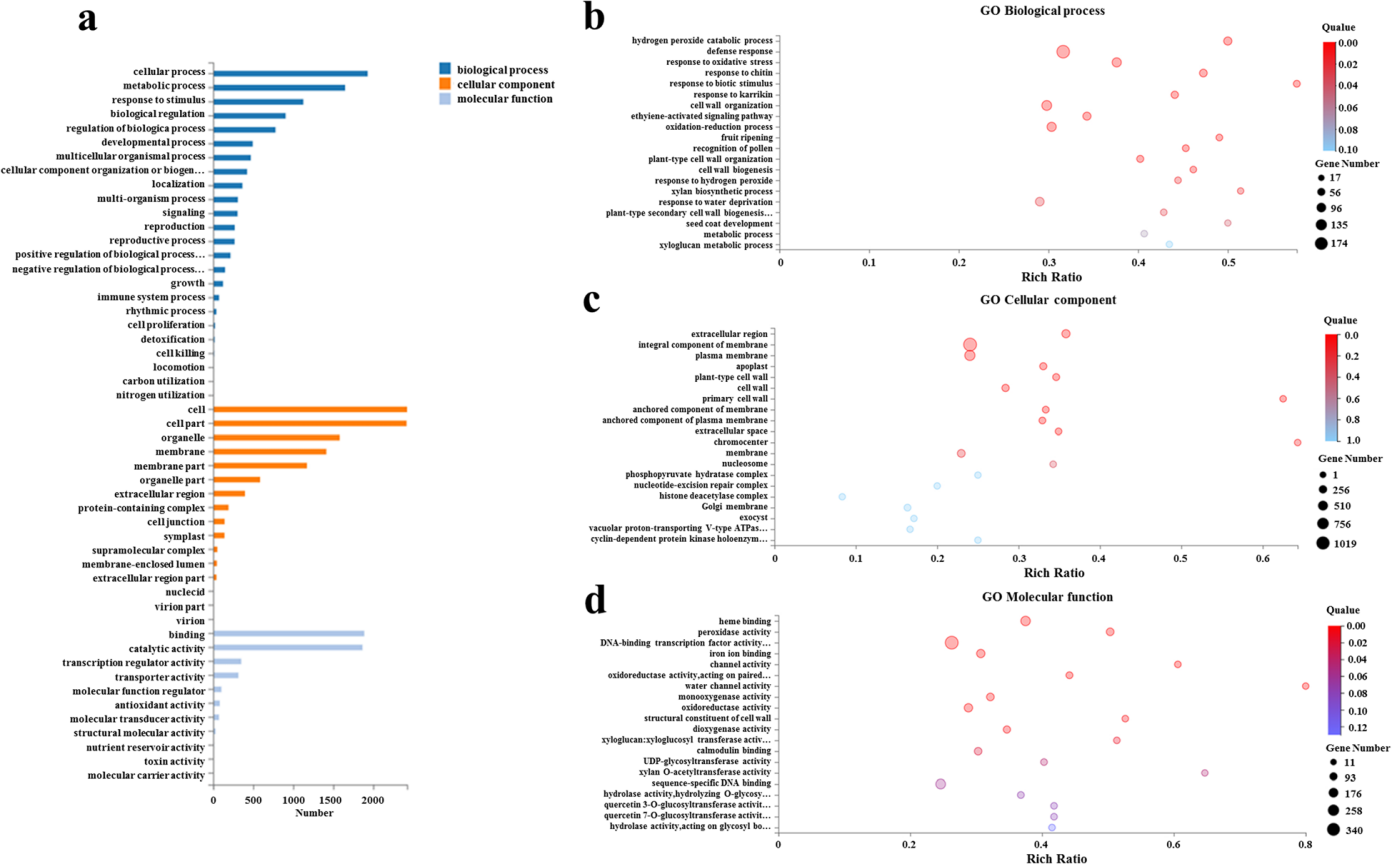
GO enrichment analysis the differentially expressed genes (DEGs) between NM and AM seedlings. **a** The significantly enriched 24 GO terms in the biological processes, 16 GO terms in the cellular components, and 11 GO terms in the molecular functions. **b** The highly enriched in the GO term of biological processes. **c** The highly enriched in the GO term of the cellular components. **d** The highly enriched in the GO term of the molecular functions

The DEGs of AM seedlings were significantly enriched for 24 GO items in biological processes, 16 GO items in cellular components, and 11 GO items in molecular function (Fig. 3a; Additional file 8: Table S5). The GO enrichment analysis results for biological processes showed that GO terms, such as hydrogen peroxide decomposition process (GO:0042744), defense response (GO:0006952), response to oxidative stress (GO:0006979), and response to biotic stimulus (GO:0009607) were enriched after AMF inoculation (Fig. 3b; Additional file 8: Table S5). The GO enrichment analysis results for cell components indicated that more significantly different genes were concentrated in the GO terms related to the cell membrane, such as extracellular region (GO:0005576), integral component of membrane (GO:0016021), plasma membrane (GO:0005886), and plant cell wall (GO:0009505) (Fig. 3c; Additional file 8: Table S5). The GO enrichment analysis results for molecular functions showed that GO items such as DNA-binding transcription factor activity (GO:0003700), peroxidase activity (GO:0004601), oxidoreductase activity (GO:0016491), iron ion binding (GO:0005506), and monooxygenase activity (GO:0004497) were enriched (Fig. 3d; Additional file 8: Table S5).

### Kyoto encyclopedia of genes and genomes (KEGG) pathway enrichment analysis

**Fig. 4:**
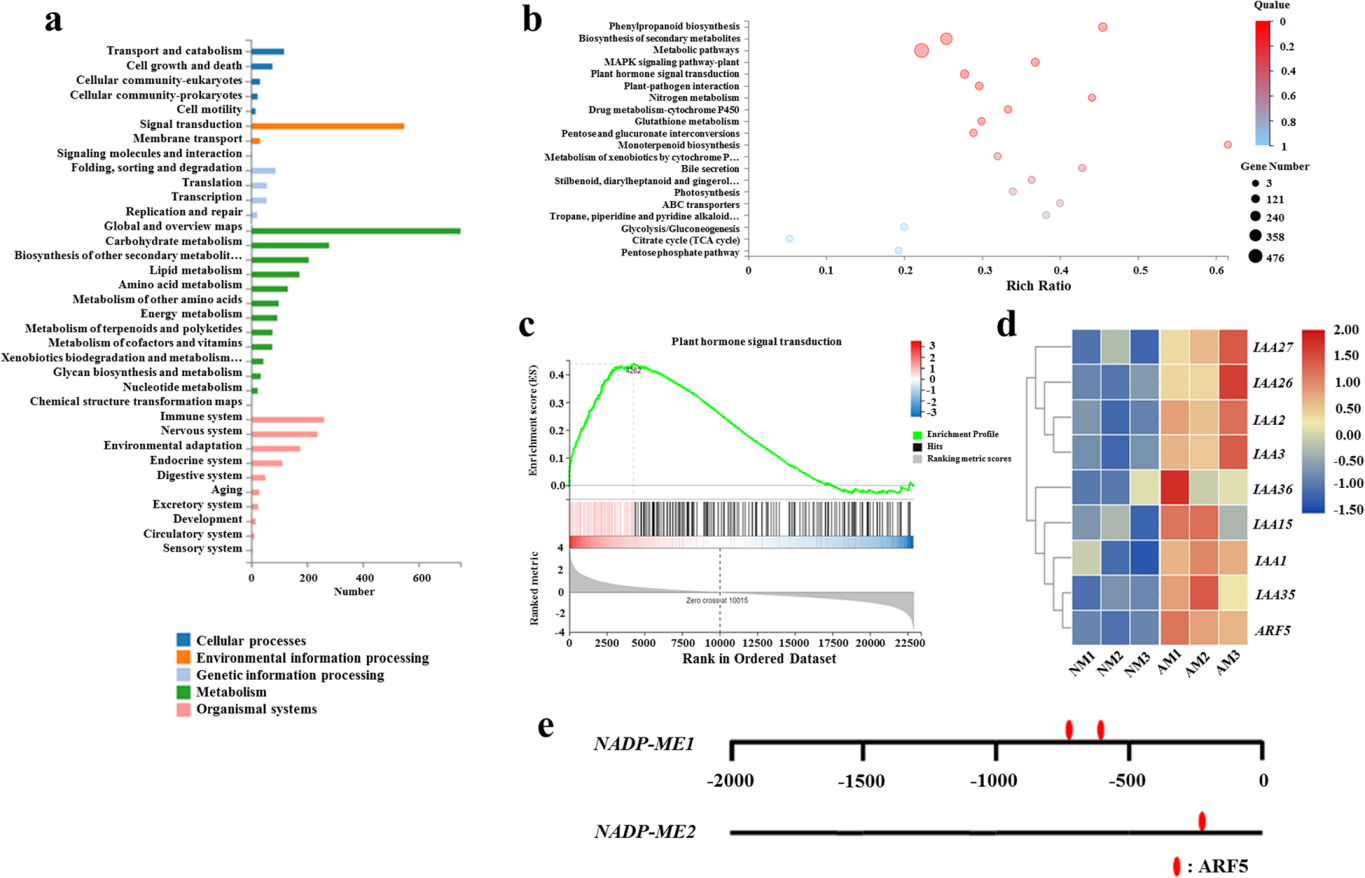
KEGG pathway and GSEA analysis the differentially expressed genes (DEGs) between NM and AM seedlings and ARF5 binding sites in *NADP-MEs* promoters. **a** KEGG pathway analysis the DEGs between NM and AM seedlings. **b** The top 20 enriched KEGG pathways. **c** GSEA enrichment analysis of genes related to plant hormone signal transduction pathway. **d** Gene heat map analysis the core enrichment genes related to IAA. **e** ARF5 binding sites in the promoters of *NADP-ME1 and NADP-ME2* of tomato. The numbering comes from the predicted transcription starting point

The DEGs were mainly enriched in 33 KEGG pathways, including cellular processes, environmental information processing, genetic information processing, metabolism, and organismal systems (Fig. 4a; Additional file 9: Table S6). The top 20 enriched KEGG pathways (Q value is arranged in ascending order) were used to draw a bubble chart (Fig. 4b; Additional file 9: Table S6). The results showed that the DEGs were enriched in phenylpropane biosynthesis (00940), biosynthesis of secondary metabolites (01110), metabolic pathways (01100), MAPK signaling pathways (04016), and plant hormone signal transduction (04075). Next, we performed gene set enrichment analysis (GSEA) using the genes related to plant signal transduction from the results of KEGG enrichment analysis (Fig. 4c; Additional file 10: Table S7) and obtained 109 genes positively related to the phenotype of AM seedlings, of which 9 genes were related to IAA (Fig. 4d). Interestingly, the GO enrichment results showed that all 9 genes were enriched in the nucleus (GO: 0005634), binding domain containing DNA (GO: 0003677), and were all enriched in the auxin activation signal pathway (GO: 0009734) and transcriptional regulation pathways (GO: 0006355). In addition, *ARF5*, an auxin regulator, was enriched in the biological process of hormone response (GO:0009725). Interestingly, the promoters of *NADP-ME1* and *NADP-ME2* contained the specific binding sites for ARF5 (Fig. 4e), indicating that ARF5 might directly regulate the expression of *NADP-ME1* and *NADP-ME2*.

### Effects of AMF on endogenous root hormone

**Fig. 5:**
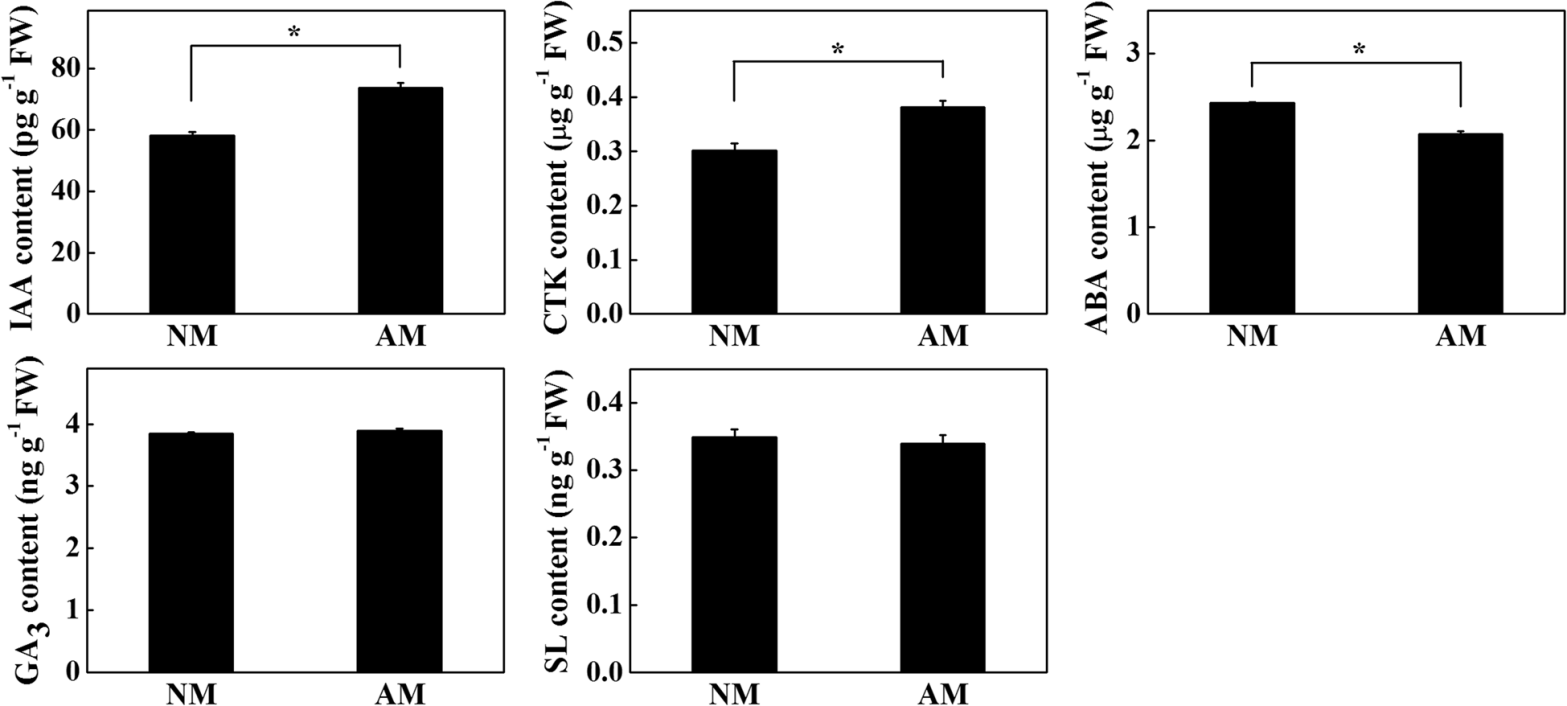
The contents of IAA, CTK, ABA, GA_3_, and SL in roots of NM and AM tomato seedlings. The results represent the means ± SE. Three independent experiments were performed, with similar results. * represent significant difference. NM, tomato seedlings cultivated in continuous cropping substrate. AM, tomato seedlings cultivated in continuous cropping substrate inoculation with AMF. FW: fresh weight

Transcriptome analysis showed that genes related to plant hormone signal transduction responded to AMF colonization. Therefore, we analyzed the concentrations of IAA, gibberellin (GA_3_), abscisic acid (ABA), cytokinin (CTK), and strigolactone (SL) in the roots of tomato seedlings. The results showed that the concentrations of IAA and CTK in the roots of AM seedlings significantly increased, while the concentrations of ABA significantly decreased, and the concentrations of GA_3_ and SL did not change compared with those in the NM seedlings (Fig. 5). These results indicated that the AMF colonization of the roots of tomato seedlings modulated endogenous hormone levels, which presumably further affected other physiological and metabolic processes.

### Response of *NADP-MEs* to hormones

To verify whether hormones mediated root activity, tomato seedlings were treated with IAA, GA_3_, ABA, CTK, and SL, and qPCR was used to test the dynamic expression patterns of *NADP-ME1* and *NADP-ME2*. The results showed that these 2 genes responded to all 5 hormones (Additional file 11: Figure S4). The expression of *NADP-ME1* and *NADP-ME2* was upregulated by IAA, and the gene expression remained highly upregulated from 1 h to 12 h after treatment, and the expression characteristics of these 2 genes were consistent (Additional file 11: Figure S4a). However, ABA significantly reduced the expression of *NADP-ME1* and *NADP-ME2* (Additional file 11: Figure S4c).

### Effects of IAA on AMF-induced root activity

**Fig. 6:**
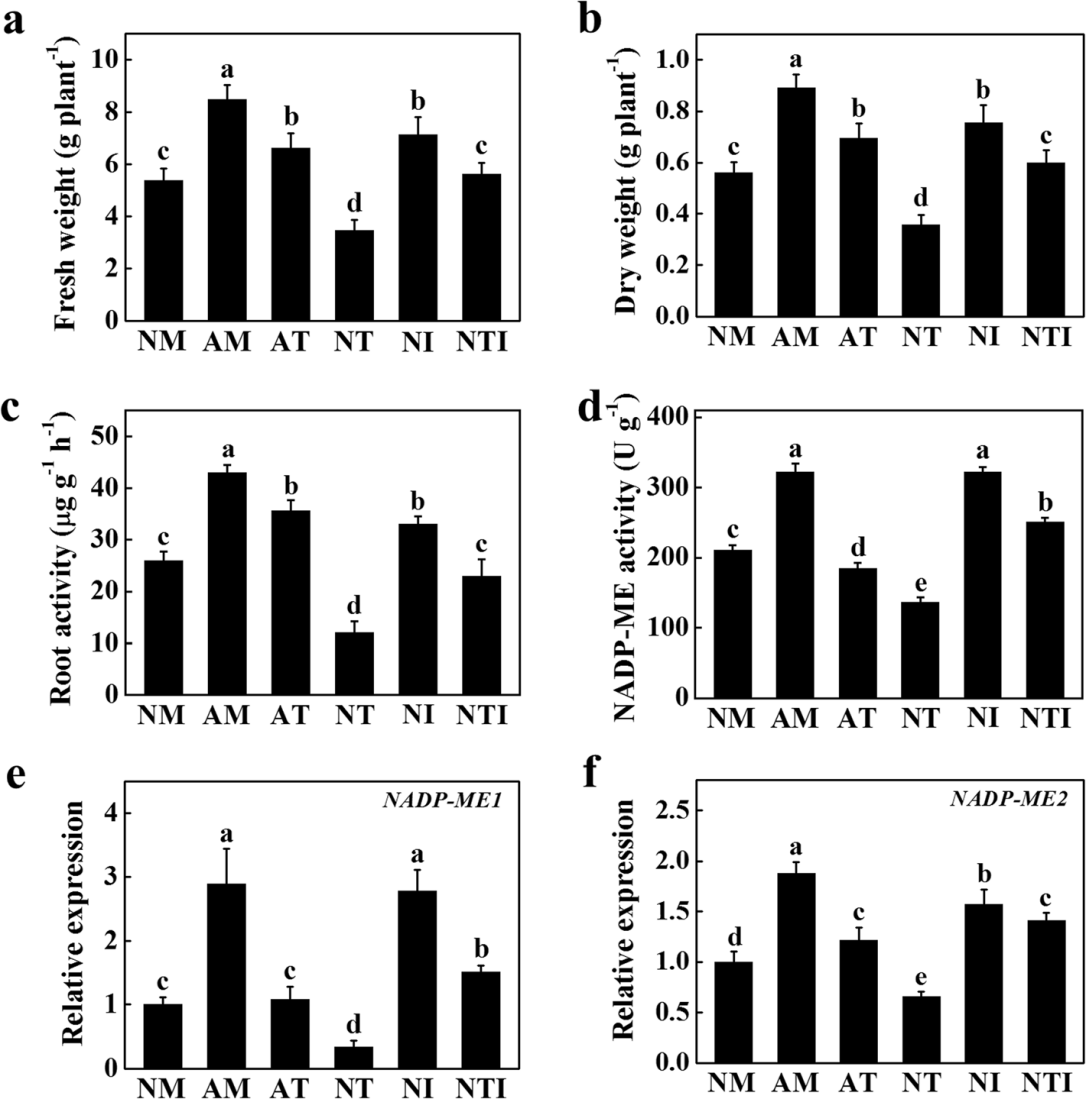
IAA mediated AMF-induced growth, root and NADP-ME activity in tomato seedlings. **a** Fresh weight. **b** Dry weight. **c** The root activity of tomato seedlings. **d** The NADP-ME activity of tomato seedlings. **e** The expression of *NADP-ME1*. **f** The expression of *NADP-ME2*. After inoculation with AMF for 15 d, IAA and TIBA were irrigated every 5 d, and the data were determined at 30 d. The results represent the means ± SE. Means with the same letter did not significantly differ at *P*< 0.05, according to Tukey’s test. Three independent experiments were performed, with similar results. NM: tomato seedlings cultivated in the continuous cropping substrate; AM: tomato seedlings cultivated in the continuous cropping substrate inoculation with AMF; AT: tomato seedlings cultivated in the continuous cropping substrate inoculation with AMF and irrigated with TIBA; NT: tomato seedlings cultivated in the continuous cropping substrate irrigated with TIBA; NI: tomato seedlings cultivated in the continuous cropping substrate irrigated with IAA; NTI: tomato seedlings cultivated in the continuous cropping substrate irrigated with TIBA, and then irrigated with IAA after 24 h

To clarify the role of IAA in AMF-induced root activity, we investigated the effects of IAA and 2,3,5-triiodobenzoic acid (TIBA, an auxin polar transport inhibitor) on root activity, NADP-ME activity, and *NADP-ME1* and *NADP-ME2* expression levels in tomato seedlings cultivated in continuous cropping substrate with or without AMF inoculation. The application of IAA significantly promoted tomato seedlings growth after 15 d of treatment, while the application of TIBA significantly inhibited the growth of tomato seedlings compared with that in the NM seedlings (Fig. 6a, b). Furthermore, the root activity of AM and tomato seedlings cultivated in the continuous cropping substrate irrigated with IAA (NI) increased by 65 and 27%, respectively, while the root activity of tomato seedlings cultivated in the continuous cropping substrate irrigated with TIBA (NT) decreased by 54% compared with that in NM seedlings (Fig. 6c). Strikingly, the root activity of tomato seedlings cultivated in the continuous cropping substrate inoculation with AMF and irrigated with TIBA (AT) was significantly lower than that in the AM seedlings (Fig. 6c). As shown in Fig. 6d, the NADP-ME activity of AT seedlings markedly decreased compared with that in the AM seedlings. The NADP-ME activity of NI seedlings significantly increased compared with that in the NM seedlings (Fig. 6d). In contrast, the NADP-ME activity of NT seedlings decreased compared with that of NM seedlings (Fig. 6d). However, the inhibitory effects of TIBA were ameliorated by the application of IAA (Fig. 6d).

Gene expression results showed that the expression of *NADP-ME1* and *NADP-ME2* in AM plants was higher than that in NM plants (Fig. 6e, f). Compared with NM and AM, TIBA significantly reduced the expression of *NADP-ME1* and *NADP-ME2* in NT and AT plants; in contrast, the application of IAA significantly increased the expression of *NADP-ME1* and *NADP-ME2* (Fig. 6e, f). The gene expression of *NADP-ME1* and *NADP-ME2* in tomato seedlings cultivated in the continuous cropping substrate irrigated with TIBA, and then irrigated with IAA after 24 h (NTI) was consistent with the trend of NADP-ME enzyme activity (Fig. 6d-f). The above results indicated that IAA mediated the expression of *NADP-ME1* and *NADP-ME2* and root activity of tomato seedlings in continuous cropping substrates.

## Discussion

### AMF promoted tomato seedling growth in continuous cropping substrate

Compared with fresh substrates, continuous cropping substrates have many limitations, such as lower pH value, decreased nutrient content, reduced permeability, and imbalanced microbial community structure [3]. Recently, the use of beneficial microorganisms has become increasingly popular to maintain soil fertility and crop productivity [21]. AMF play a critical role in plant response to biotic and abiotic stresses [15, 22–24]. AMF can reduce the risk of ionic toxicity and cell membrane damage by decreasing Na^+^ absorption and increasing antioxidant enzyme activity in cucumber [25]. Studies have shown that AMF significantly improve the physicochemical properties and enzyme activities of continuous cropping systems and promote the absorption of nutrients, resulting in promoted plant growth and increased yield [15, 16, 26]. Consistent with the previous studies, AMF incorporation into the continuous cropping substrate remarkably improved the growth of tomato seedlings and enhanced the yield of tomato (Fig. 1; Additional file 3: Figure S2). These results indicate that the inoculation of AMF in continuous cropping substrates is an effective way to relieve continuous cropping obstacles.

### AMF induced an increase in endogenous IAA in tomato roots

It has been shown that the inoculation of AMF can regulate hormone levels in plants [27, 28]. In this study, the inoculation of AMF significantly increased IAA and CTK concentrations in tomato roots and significantly reduced ABA concentrations (Fig. 5). Similarly, AMF increased the levels of IAA and CTK, decreased ABA levels, and enhanced hormone homeostasis in the damaged roots of maize [29]. Furthermore, AMF inoculation significantly increased the IAA content in the root system of trifoliate orange and decreased IAA loss in the root system [30]. In this study, the enrichment analysis of the KEGG pathway showed that the plant hormone signal transduction pathway was relatively enriched (Fig. 4b). Through GSEA, 109 genes were positively correlated with the AM phenotype, among which 9 genes were correlated with IAA (Fig. 4c, d). Similarly, AMF can increase the IAA level and promote the growth and development of host plants under stress by upregulating the expression of IAA transporter genes and downregulating the expression of auxin efflux genes [31]. In addition, AMF have a positive effect on the regulation of IAA levels in plants under drought [32], salt stress [33] and biotic stress [34]. These results suggested that the increase in IAA levels in tomato roots induced by AMF might be an important reason to promote the growth and development of tomato seedlings in continuous cropping substrates.

### IAA mediated AMF-induced *NADP-MEs* expression and NADP-ME activity

NADP-ME is a cytoplasmic protein that is expressed during the development of tomato roots, stems, leaves, and fruits [35]. In particular, *NADP-ME1* is involved in the regulation of malic acid content in the root apex, and the absence of *NADP-ME1* results in an increase in malic acid content. Furthermore, *NADP-ME1* also affects the signal transmission process [36]. However, *NADP-ME2* plays an important role in the defense mechanism of plants and seems to be involved in the production of reactive oxygen species [37]. Our results showed that *NADP-ME1* and *NADP-ME2* responded to IAA, GA_3_, ABA, CTK, and SL (Additional file 11: Figure S4). Among them, IAA significantly increased the expression of *NADP-ME1* and *NADP-ME2* (Additional file 11: Figure S4a), which indicated that the induction of *NADP-ME1* and *NADP-ME*2 by IAA might be a response to the stress of tomato under continuous cropping substrates. Notably, hormones play a crucial role in regulating complex signaling networks in different plant growth and development processes and in plant responses to environmental stresses [38]. Likewise, IAA is involved in regulating the defense response to various biotrophic and necrotrophic pathogens [38]. Our results showed that AMF inoculation significantly increased the IAA concentration in tomato roots (Fig. 5). Furthermore, IAA application significantly increased the expression levels of *NADP-ME1* and *NADP-ME2*, NADP-ME activity, root activity, and the growth of tomato seedlings cultivated in continuous cropping substrates (Fig. 6). However, these effects were compromised when plants were treated with TIBA (Fig. 6). Similarly, AMF inoculation promotes the accumulation of endogenous IAA to improve the root system and nutrient absorption under stress conditions [39]. Exogenous IAA significantly increases the activity of NADP-ME in *Malus baccata* (L.) Borkh. [40]. Thus, AMF inoculation improved the endogenous IAA level in plant roots to increase the expression of *NADP-ME1* and *NADP-ME2* and NADP-ME activity.

## Conclusions

In conclusion, our study revealed that IAA mediates the AMF-promoted tomato growth and *NADP-MEs* expression in continuous cropping substrates. Our study suggests that the use of AMF can effectively improve the growth of tomato cultivated in continuous cropping substrates, thereby showing a promising solution for the reuse of continuous cropping substrates with AMF.

## Methods

### Plant materials and treatments

Tomato (*Solanum lycopersicum* L. cv hezuo 903, obtained from Shanghai Changzhong Tomato Seed Industry Co., Ltd.) was used in this study. The AMF used in this study was an isolate of *Funneliformis mosseae* (BGC HEB07B, 1511C0001BGCAM0049, obtained from Huaian Chaimihe Agriculture Science and Technology Co., Ltd.), which was obtained through propagation with maize (*Zea mays* L.) as previously described [41].

The continuous cropping substrate, which had been used for the cultivation (complete growth cycle) of pepper and eggplant, was sterilized and then used to cultivate tomato in this study. The physical properties of the continuous cropping substrate deteriorated, the bulk density (BD) significantly increased, and the values of pH and electrical conductivity (EC) decreased (Additional file 12: Table S8).

The tomato seeds were placed on moist filter paper and germinated at 28 °C for 30 h in the dark. After germination, the seeds were sown in plastic pots containing continuous cropping substrate or continuous cropping substrate inoculated with *F. mosseae* at a dose of 600 spores per plant. Seedlings were cultivated in an artificial growth chamber. The growth conditions were as follows: 25 ± 2 °C/18 ± 2 °C (day/night), 12 h/12 h (day/night) photoperiod, 60–75% relative humidity, and 300 μmol m^− 2^ s^− 1^ light density. The growth parameters, root morphology, and Pn of tomato seedlings were determined at 40 d. The enzyme activity related to root activity was measured at 20, 25, 30, 35, and 40 d. The transcriptome sequencing was performed at 30 d with 3 biological replicates.

### Hormone treatments

Tomato seedlings were cultured in fresh substrate and grown under in the same conditions as described above. The tomato seedlings were watered with 50 ml of 100 μM GA_3_ (Solarbio, Beijing, China), IAA (Solarbio, Beijing, China), ABA (Solarbio, Beijing, China), CTK (Solarbio, Beijing, China), and SL (Solarbio, Beijing, China) solution per plant, respectively, and the control plants were watered with the same volume of deionized water. Root samples were taken at 0, 1, 6, 12, and 24 h, respectively, to determine the gene expression of *NADP-MEs*.

### IAA and TIBA treatment

To clarify the relationship between IAA and tomato root activity and *NADP-MEs*, 15-d-old NM and AM tomato seedlings were watered with 50 ml of 100 μM IAA or TIBA (Aladdin, Shanghai, China), every 5 d, and the control plants were watered with the same volume of deionized water. There were 6 treatments in the experiment, including NM, AM, AT, NT, NI, and NTI. Root samples were taken at 30 d to determine the root activity, enzyme activities, and *NADP-MEs* expression.

### Mycorrhizal colonization rate measurement

For measurement of the rate of mycorrhizal colonization, fresh roots were collected at 30 d, cleaned and cut to 1–2 cm and then incubated in 10% (w/v) KOH (Sinopharm Chemical Reagent, Shanghai, China) at 90 °C for 40 min. The roots were rinsed with distilled water and then soaked in 2% lactic acid (Solarbio, Beijing, China) at 90 °C for 20 min. Then, the roots were stained with 0.05% Trypan Blue dye (Solarbio, Beijing, China) at 90 °C for 30 min. After cooling, the root samples were decolored with destaining solution (distilled water: lactic acid: glycerin [Sinopharm Chemical Reagent, Shanghai, China] =1:2:2, v/v) for 2–4 d at room temperature. Thirty pieces of randomly selected stained root fragments were observed with a Leica DM1000 microscope (Leica Microsystems, Wetzlar, Hesse, Germany) to confirm the presence of fungal structures, including intraradical mycelia, vesicles, and arbuscules. The root colonization rate was measured as previously described [42].

### Morphological index and Pn measurement

The plant height and stem diameter were measured at 40 d according to previously described methods [43, 44]. After removing the plants from the cultivation pot, the roots were washed with distilled water and the fresh weight was measured with an electronic balance (OHAUS, Parsippany, NJ, USA). The plant materials were enclosed in envelopes and placed in an oven (Shanghai Yiheng Scientific Instrument Co., Ltd., Shanghai, China) at 105 °C for 30 min. Then, the oven temperature was adjusted to 75 °C for 2 d to obtain the dry weight. A WinRHIZO LA2400 root scanner system (Regent Instruments Inc., Québec, QC, Canada) was used to collect root morphological indexes.

The Pn was measured with a portable photosynthesis measurement system (Li-6400; Li-COR, Lincoln, NE, USA) after 1 h of light in the morning.

### Root activity and related enzyme activities

The root activity of tomato was determined with the triphenyltetrazolium chloride (TTC) method as previously described [45].

The activities of 6PGDH, G6PDH, NADP-IDCH and NADP-ME were measured by spectrophotometry, recording the reduction of NADP at 340 nm [46, 47]. The experiment was conducted at 25 °C, and the reaction system volume was 1 ml, including 50 mM HEPES (pH 7.6, Solarbio, Beijing, China), 2 mM MgCl_2_ (Sinopharm Chemical Reagent, Shanghai, China), 0.8 mM NADP (Solarbio, Beijing, China) and plant samples. The reaction was initiated by adding 5 mM 6-phosphate gluconate (Aladdin, Shanghai, China), 5 mM glucose 6-phosphate (Solarbio, Beijing, China), 10 mM 2R,3S-isocitrate (Aladdin, Shanghai, China) and 10 mM malic acid (Solarbio, Beijing, China), respectively.

### Analysis of the contents of IAA, GA_3_, CTK, ABA, and SL in tomato roots

The hormone contents were determined at 30 d as previously described [48]. Tomato root tissue was fully ground in liquid nitrogen, and transferred to a precooled 50-ml centrifuge tube that contained 4 ml of precooled 80% chromatographic methanol (Aladdin, Shanghai, China). The mixture was placed on ice in the dark for 12 h. The tubes were centrifuged at 10000 *g* and 4 °C for 15 min, and the supernatant was transferred to another 50-ml tube and stored in a refrigerator at 4 °C. Afterward, 3 ml of 80% methanol was added to the remaining precipitate 3 times, and the supernatants were combined. Then, 1.0 g of polyvinylpyrrolidone (Sinopharm Chemical Reagent, Shanghai, China) was added to the supernatant and shaken in a shaker at 4 °C in the dark for 1 h. Subsequently, the mixture was centrifuged at 10000 *g* and 4 °C for 15 min, and the supernatant was passed through a C18 extraction cartridge (Waters, Milford, MA, USA) that had already been rinsed in the dark. The liquid was stored in a 50-ml centrifuge tube and freeze-dried in vacuum for 3 d under dark conditions. Then, 1 ml of precooled chromatographic methanol was added to the tube to completely dissolve the hormone, and the samples were filtered with a 0.45-μm organic microfiltration membrane before loading. The samples were detected with a high-performance liquid chromatography 1525 system (Waters, Milford, MA, USA).

### qPCR analysis

Total RNA was isolated from tomato roots with the RNA Simple Total RNA Kit (Tiangen, Beijing, China). Total RNA (1 μg) was reverse transcribed into cDNA using HiScript® II Q RT SuperMix (+ gDNA-wiper) (Vazyme, Nanjing, China) for qPCR. qPCR assays were performed using ChamQ Universal SYBR-qPCR Master Mix (Vazyme, Nanjing, China) in a StepOne (TM) real-time PCR system (Applied Biosystems, Foster, CA, USA). The tomato *Ubi3* gene was used as an internal control. The primer sequences are shown in Additional file 13: Table S9. The relative gene expression was calculated as described by Livak and Schmittgen [49].

### RNA extraction, cDNA library construction and Illumina sequencing

Total RNA was extracted from the roots of tomato seedlings using TRIzol Reagent (Invitrogen, Carlsbad, CA, USA). The RNA quality and purity were verified by a Nanodrop 2000 (Thermo Fisher Scientific, Rockford, IL, USA) and electrophoresis in a 1.0% agarose gel. The mRNAs were purified from total RNA using poly-T oligo-attached magnetic beads (Invitrogen, Carlsbad, CA, USA). Subsequently, the mRNAs were fragmented, and cDNA was synthesized using random hexamers, DNA polymerase I (Thermo Fisher Scientific, Rockford, IL, USA) and RNase H (Thermo Fisher Scientific, Rockford, IL, USA). The purified double-stranded cDNAs were ligated to adaptors for Illumina paired-end sequencing. An Agilent 2100 Bioanalyzer (Agilent Technologies, Santa Clara, CA, USA) and ABI real-time RT-PCR (Applied Biosystems, Foster, CA, USA) were used to verify the quality and quantity of the library, respectively. The cDNA libraries were sequenced with the Illumina HiSeq2000 platform (Illumina, San Diego, CA, USA) by the Beijing Genomics Institute.

### Sequence data analysis and annotation

After the raw reads, adaptor sequences and low-quality reads were removed, all the clean reads were mapped to the tomato reference genome using TopHat v1.4.0 [50]. The transcript abundance was normalized by the fragments per kilobase of exon per million fragments mapped using Cufflinks [51].

### Identification of DEGs

The significance of the gene expression difference was recognized based on the false discovery rate (FDR) value less than 0.01 and |log_2_(fold change)| ≥ 2. After normalization, hierarchical clustering and k-means clustering analysis of the expression patterns were performed using Mutiexperimental Viewer v4.7 [52].

### GO and KEGG enrichment analysis

For identification of putative biological functions and pathways of the DEGs, the GO and KEGG database were searched for annotation. GO classification was performed by WEGO [53]. The AgriGO and KOBAS2.0 packages were used to analyze the enrichment of GO and KEGG at a significance cutoff of 0.05 FDR, respectively [54, 55].

### Statistical analysis

The experiment was carried out in a completely randomized design with three independent replicates, and each replicate contained 12 plants. Significant differences (*P*< 0.05) between treatments were determined using Tukey’s test.

## Supplementary Information

**Additional file 1: Figure S1.** The colonization of AMF in tomato seedlings.

**Additional file 2: Table S1.** Effects of AMF inoculation on tomato root growth.

**Additional file 3: Figure S2.** Effects of arbuscular mycorrhizal fungi (AMF) inoculation on the yield of tomato.

**Additional file 4: Table S2.** Base statistics after filtering from NM1, NM2, NM3, AM1, AM2 and AM3 libraries.

**Additional file 5: Table S3.** Mapping results of clean reads against the tomato genome and gene.

**Additional file 6: Figure S3.** Gene heat map showing the gene expression differences between NM and AM seedling roots.

**Additional file 7: Table S4.** The differentially expressed genes between NM and AM.

**Additional file 8: Table S5.** GO enrichment analysis the differentially expressed genes between NM and AM.

**Additional file 9: Table S6.** KEGG pathway analysis the differentially expressed genes between NM and AM.

**Additional file 10: Table S7.** GSEA enrichment analysis of genes related to plant hormone signal transduction pathway.

**Additional file 11: Figure S4.** Responses of *NADP-ME1* and *NADP-ME2* to hormones.

**Additional file 12: Table S8.** Comparison of physical and chemical properties of continuous cropping substrate and fresh substrate.

**Additional file 13: Table S9.** Primers used for qPCR assays.

## Data Availability

The data charts supporting the results and conclusions are included in the article and additional files. All sequences generated by sequencing for this study are available in the NCBI Sequence Read Archive (SRA) database under Bioproject PRJNA686719 (http://www.ncbi.nlm.nih.gov/bioproject/686719). The materials are available upon request by contacting the corresponding author.

## Abbreviations

6PGDH: 6-phosphate gluconate dehydrogenase
ABA: Abscisic acid
AM: Tomato seedlings cultivated in the continuous cropping substrate inoculation with AMF
AMF: Arbuscular mycorrhizal fungi
AT: Tomato seedlings cultivated in the continuous cropping substrate inoculation with AMF and irrigated with TIBA
CTK: Cytokinin
DEGs: Differentially expressed genes
EC: Electrical conductivity
FDR: False discovery rate
FW: Fresh weight
G6PDH: Glucose-6-phosphate dehydrogenase
GA_3_: Gibberellin
GO: Gene ontology
GSEA: Gene set enrichment analysis
IAA: Indole acetic acid
KEGG: Kyoto encyclopedia of genes and genomes
NADP-IDCH: NADP-isocitrate dehydrogenase
NADP-ME: NADP-malic enzyme
NI: Tomato seedlings cultivated in the continuous cropping substrate irrigated with IAA
NM: Tomato seedlings cultivated in the continuous cropping substrate
NT: Tomato seedlings cultivated in the continuous cropping substrate irrigated with TIBA
NTI: Tomato seedlings cultivated in the continuous cropping substrate irrigated with TIBA, and then irrigated with IAA after 24 h
Pn: Net photosynthetic rate
SL: Strigolactone
TIBA: 2,3,5-triiodobenzoic acid
