# Supplementary material for: Auxin is involved in arbuscular mycorrhizal fungi-promoted tomato growth and NADP-malic enzymes expression in continuous cropping substrates
Source: BMC Plant Biol. 2021 Jan 18;21:48. doi: 10.1186/s12870-020-02817-2 (PMC7814736; doi:10.1186/s12870-020-02817-2)
Supplement: Supplementary file 1 — Additional file 1: Figure S1. The colonization of AMF in tomato seedlings. [file 12870_2020_2817_MOESM1_ESM.pdf]

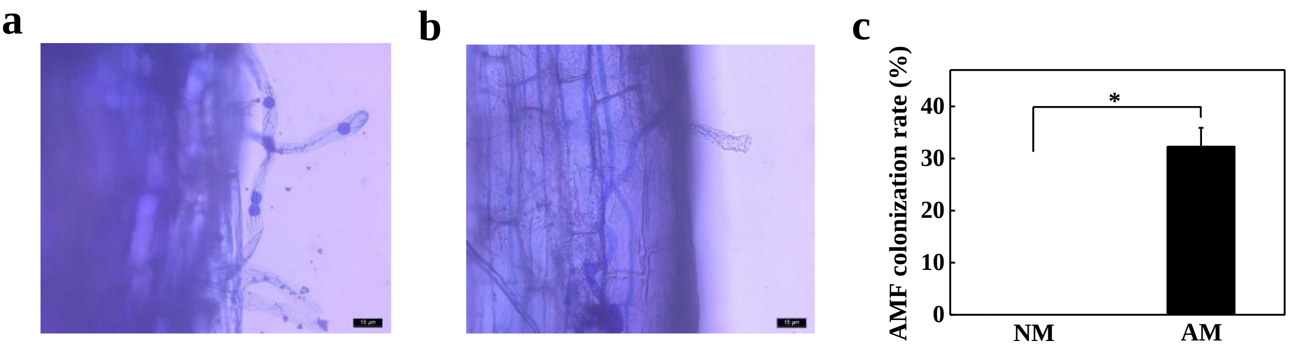

Fig. S1. The colonization of AMF in tomato seedlings. **a** and **b** Representative images of AMF colonization in tomato roots. **c** AMF colonization rate. Tomato seedlings cultivated in continuous cropping substrate with AMF inoculation, and the roots were collected at 30 d to detect the colonization. The results represent the means ± SE. Three independent experiments were performed, with similar results. * represent significant difference. NM, tomato seedlings cultivated in continuous cropping substrate. AM, tomato seedlings cultivated in continuous cropping substrate inoculation with AMF.
