# Supplementary material for: Auxin is involved in arbuscular mycorrhizal fungi-promoted tomato growth and NADP-malic enzymes expression in continuous cropping substrates
Source: BMC Plant Biol. 2021 Jan 18;21:48. doi: 10.1186/s12870-020-02817-2 (PMC7814736; doi:10.1186/s12870-020-02817-2)
Supplement: Supplementary file 2 — Additional file 2: Table S1. Effects of AMF inoculation on tomato root growth. [file 12870_2020_2817_MOESM2_ESM.docx]

**Table S1.** Effects of AMF inoculation on tomato root growth.

| Treatments | Root length (mm) | Total root surface area (cm^2^) | Total root volume (cm^3^) | Average root diameter (mm) | Number of root tips | Root fresh weight (g) | Root dry weight (g) |
| --- | --- | --- | --- | --- | --- | --- | --- |
| NM | 1856.85±56.01 | 273.74±12.67 | 3.49±0.21 | 0.49±0.01 | 5095±339.33 | 1.40±0.08 | 0.13±0.01 |
| AM | 2445.09±21.92* | 354.98±3.86* | 4.30±0.08* | 0.52±0.01 | 6490±89.40* | 2.69±0.05* | 0.20±0.02* |

All results represent the means ± SE. Three independent experiments were performed with similar results. * represent significant difference. NM, tomato seedlings cultivated in continuous cropping substrate. AM, tomato seedlings cultivated in continuous cropping substrate inoculation with AMF.
