# Supplementary material for: Auxin is involved in arbuscular mycorrhizal fungi-promoted tomato growth and NADP-malic enzymes expression in continuous cropping substrates
Source: BMC Plant Biol. 2021 Jan 18;21:48. doi: 10.1186/s12870-020-02817-2 (PMC7814736; doi:10.1186/s12870-020-02817-2)
Supplement: Supplementary file 3 — Additional file 3: Figure S2. Effects of arbuscular mycorrhizal fungi (AMF) inoculation on the yield of tomato. [file 12870_2020_2817_MOESM3_ESM.pdf]

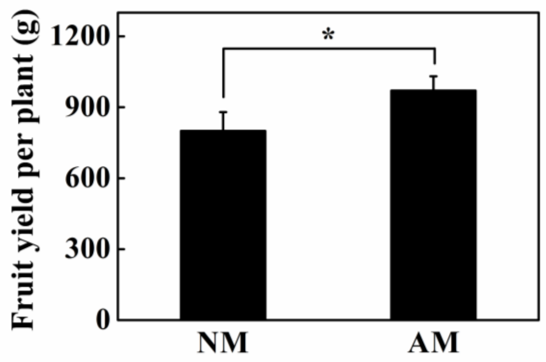

Fig. S2. Effects of arbuscular mycorrhizal fungi (AMF) inoculation on the yield of tomato. The results represent the means ± SE. Three independent experiments were performed, with similar results. * represent significant difference. NM, tomato seedlings cultivated in continuous cropping substrate. AM, tomato seedlings cultivated in continuous cropping substrate inoculation with AMF.
