# Supplementary material for: Auxin is involved in arbuscular mycorrhizal fungi-promoted tomato growth and NADP-malic enzymes expression in continuous cropping substrates
Source: BMC Plant Biol. 2021 Jan 18;21:48. doi: 10.1186/s12870-020-02817-2 (PMC7814736; doi:10.1186/s12870-020-02817-2)
Supplement: Supplementary file 4 — Additional file 4: Table S2. Base statistics after filtering from NM1, NM2, NM3, AM1, AM2 and AM3 libraries. [file 12870_2020_2817_MOESM4_ESM.docx]

**Table S2.** Base statistics after filtering from NM1, NM2, NM3, AM1, AM2 and AM3 libraries.

| Sample name | Clean read number | Clean bases (G) | Q20 (%) | Q30(%) | Clean reads ratio (%) |
| --- | --- | --- | --- | --- | --- |
| NM1 | 68867210 | 6.89 | 98.06 | 91.48 | 91.87 |
| NM2 | 64910610 | 6.49 | 98.09 | 91.59 | 92.78 |
| NM3 | 64905228 | 6.49 | 98.09 | 91.55 | 92.77 |
| AM1 | 67022360 | 6.70 | 97.98 | 91.17 | 92.49 |
| AM2 | 66929776 | 6.69 | 98.09 | 91.56 | 92.36 |
| AM3 | 64891092 | 6.49 | 98.25 | 92.14 | 92.75 |
