# Supplementary material for: Auxin is involved in arbuscular mycorrhizal fungi-promoted tomato growth and NADP-malic enzymes expression in continuous cropping substrates
Source: BMC Plant Biol. 2021 Jan 18;21:48. doi: 10.1186/s12870-020-02817-2 (PMC7814736; doi:10.1186/s12870-020-02817-2)
Supplement: Supplementary file 5 — Additional file 5: Table S3. Mapping results of clean reads against the tomato genome and gene. [file 12870_2020_2817_MOESM5_ESM.docx]

**Table S3.** Mapping results of clean reads against the tomato genome and gene.

| Sample name | Genome | | Gene | |
| --- | --- | --- | --- | --- |
|  | Mapping genome ratio (%) | Uniquely mapping genome ratio (%) | Mapping gene ratio (%) | Uniquely mapping gene ratio (%) |
| NM1 | 93.02 | 91.40 | 83.96 | 78.05 |
| NM2 | 92.58 | 90.90 | 83.91 | 78.27 |
| NM3 | 93.01 | 91.31 | 84.67 | 78.74 |
| AM1 | 93.12 | 91.18 | 83.68 | 77.21 |
| AM2 | 93.18 | 91.11 | 83.79 | 76.93 |
| AM3 | 94.76 | 92.60 | 84.45 | 77.69 |
