# Supplementary material for: Auxin is involved in arbuscular mycorrhizal fungi-promoted tomato growth and NADP-malic enzymes expression in continuous cropping substrates
Source: BMC Plant Biol. 2021 Jan 18;21:48. doi: 10.1186/s12870-020-02817-2 (PMC7814736; doi:10.1186/s12870-020-02817-2)
Supplement: Supplementary file 6 — Additional file 6: Figure S3. Gene heat map showing the gene expression differences between NM and AM seedling roots. [file 12870_2020_2817_MOESM6_ESM.pdf]

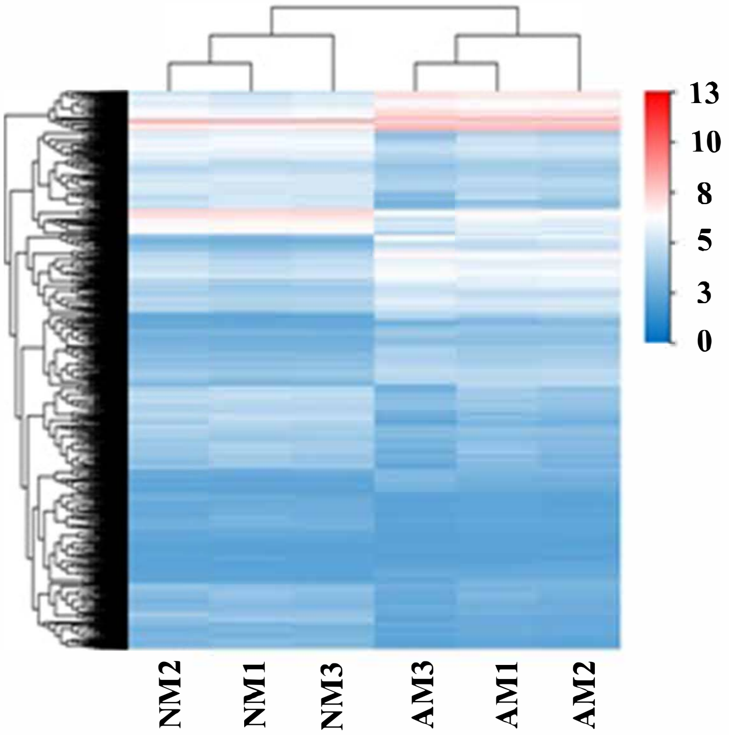

Fig. S3. Gene heat map showing the gene expression differences between NM and AM seedling roots. NM, tomato seedlings cultivated in continuous cropping substrate. AM, tomato seedlings cultivated in continuous cropping substrate inoculation with AMF.
