# Supplementary material for: Auxin is involved in arbuscular mycorrhizal fungi-promoted tomato growth and NADP-malic enzymes expression in continuous cropping substrates
Source: BMC Plant Biol. 2021 Jan 18;21:48. doi: 10.1186/s12870-020-02817-2 (PMC7814736; doi:10.1186/s12870-020-02817-2)
Supplement: Supplementary file 11 — Additional file 11: Figure S4. Responses of NADP-ME1 and NADP-ME2 to hormones. [file 12870_2020_2817_MOESM11_ESM.pdf]

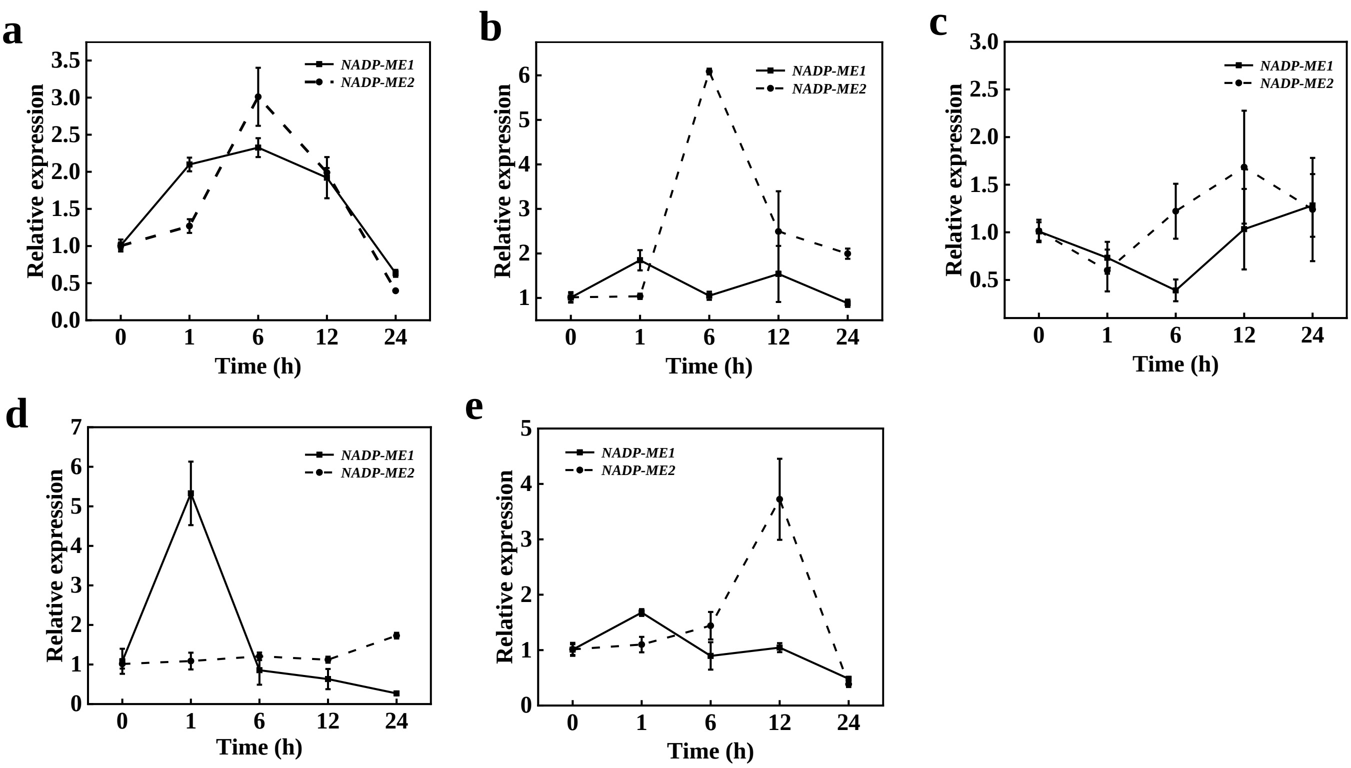

Fig. S4. Responses of *NADP-ME1* and *NADP-ME2* to hormones. **a** Effects of IAA on the expression of *NADP-ME1* and *NADP-ME2*. **b** Effects of GA_3 on the expression of *NADP-ME1* and *NADP-ME2*. **c** Effects of ABA on the expression of *NADP-ME1* and *NADP-ME2*. **d** Effects of CTK on the expression of *NADP-ME1* and *NADP-ME2*. **e** Effects of SL on the expression of *NADP-ME1* and *NADP-ME2*. The results represent the means ± SE. Three independent experiments were performed, with similar results.
