# Supplementary material for: Auxin is involved in arbuscular mycorrhizal fungi-promoted tomato growth and NADP-malic enzymes expression in continuous cropping substrates
Source: BMC Plant Biol. 2021 Jan 18;21:48. doi: 10.1186/s12870-020-02817-2 (PMC7814736; doi:10.1186/s12870-020-02817-2)
Supplement: Supplementary file 12 — Additional file 12: Table S8. Comparison of physical and chemical properties of continuous cropping substrate and fresh substrate. [file 12870_2020_2817_MOESM12_ESM.docx]

**Table S8.** Comparison of physical and chemical properties of continuous cropping substrate and fresh substrate.

| Material | BD (g cm^-3^) | TP (%) | AP (%) | WHP (%) | pH | EC (mS cm^-1^) |
| --- | --- | --- | --- | --- | --- | --- |
| Fresh substrate | 0.5871±0.01 | 87.05±3.20 | 5.79±0.83 | 81.25±5.10 | 5.31±0.059* | 1.96±0.020* |
| Continuous cropping substrate | 0.7128±0.02* | 80.66±5.66 | 3.44±0.77 | 81.15±8.65 | 4.71±0.081 | 1.19±0.067 |

BD, bulk density; TP, total porosity; AP, aeration porosity; WHP, water holding porosity; EC, electrical conductivity. * represent significant difference.
