# Supplementary material for: Auxin is involved in arbuscular mycorrhizal fungi-promoted tomato growth and NADP-malic enzymes expression in continuous cropping substrates
Source: BMC Plant Biol. 2021 Jan 18;21:48. doi: 10.1186/s12870-020-02817-2 (PMC7814736; doi:10.1186/s12870-020-02817-2)
Supplement: Supplementary file 13 — Additional file 13: Table S9. Primers used for qPCR assays. [file 12870_2020_2817_MOESM13_ESM.docx]

**Table S9.** Primers used for qPCR assays.

| Primer | Sequence (5’ 3’) |
| --- | --- |
| NADP-ME1-F | TGGATGGTGGATTCTAAGGGG |
| NADP-ME1-R | GACTGTGATGTTGGATTGGAG |
| NADP-ME2-F | TGAGAAGTTATTGAACGACGAG |
| NADP-ME2-R | TGCGAGGTACGATATTTAGCC |
| Ubi3-F | TGGTCGGAATGGGACAGAAG |
| Ubi3-R | CTCAGTCAGGAGAACAGGGT |
